# Peripheral blood lymphocytes from low-grade squamous intraepithelial lesions patients recognize vaccine antigens in the presence of activated dendritic cells, and produced high levels of CD8 + IFNγ + T cells and low levels of IL-2 when induced to proliferate

**DOI:** 10.1186/1750-9378-7-12

**Published:** 2012-05-29

**Authors:** Jorge Hernández-Montes, Leticia Rocha-Zavaleta, Alberto Monroy-García, Benny Weiss-Steider, María del Carmen Zaragoza-Ortega, Fernando Cruz-Talonia, Omar Cruz y Cruz, Laura Bonifaz-Alfonso, Adriana Karina Chávez-Rueda, Martha Patricia Rojo-Aguilar, María Victoria Legorreta-Haquet, María de Lourdes Mora-García

**Affiliations:** 1 Laboratorio de Inmunobiología, Unidad de Investigación en Diferenciación Celular y Cáncer. FES-Zaragoza, UNAM, México, Laboratorio 3, PB, UMIEZ. Campus II. Facultad de Estudios Superiores Zaragoza, UNAM, Batalla 5 de mayo s/n, Col. E. de Oriente, Esquina Fuerte de Loreto, Iztapalapa, CP 09230, México, DF, Mexico; 2 Departamento de Biología Molecular y Biotecnología, Inst. de Investigaciones Biomédicas, UNAM, Mexico; 3 Clínica de Colposcopía, Fundación Cruz-Talonia, México, DF, Mexico; 4 Unidad de Investigación Médica en Enfermedades Autoinmunes. IMSS, CMN SXXI, México, Mexico; 5 Unidad de Investigación en Inmunología, Hospital de Pediatría, IMSS, CMN SXXI, México, Mexico; 6 Unidad de Investigación Médica en Enfermedades Oncológicas. IMSS, CMN SXXI, México, Mexico

**Keywords:** Peripheral blood lymphocytes, HPV, LSIL, IL-2 deficiency

## Abstract

**Background:**

Most infections with human papillomavirus (HPV) are resolved without clinical intervention, but a minority evolves into chronic lesions of distinct grades, including cervical-uterine cancer. It is known that in most cases the immune system mediates elimination of HPV infection. However, the mechanism of immune evasion leading to HPV persistence and development of early cervical lesions is not fully understood. The aim of the present work was to evaluate the potential of peripheral blood leukocytes (PBL) from low-grade squamous intraepithelial lesions (LSIL) patients to be activated *ex-vivo* by vaccine antigens, the participation of cytotoxic lymphocytes and regulatory T cells, and to determine the secretion of Th1 and Th2 cytokines mediated by stimulation of T cell receptors.

**Results:**

We found that PBL from LSIL patients showed a significantly lower proliferation rate to vaccine antigens as compared to that of healthy donors, even though there was not a difference in the presence of antibodies to those antigens in sera from both groups. We did not find differences in either the frequency of CD4 + CD25 + FoxP3+ in PBL, or the levels of IL-4, IL-5 and IL-10 in plasma or conditioned media from PBL incubated with TcR agonists *in vitro*, between the two groups. However, we detected a lower production of IL-2 and a higher proportion of CD8 + IFNγ + cells in PBL from LSIL patients as compared with PBL from normal donors. We also observed that PBL from patients infected by HPV-16 and −18 were not able to proliferate in the presence of soluble HPV antigens added to the culture; however, a high level of proliferation was attained when these antigens were presented by activated dendritic cells.

**Conclusions:**

Our results suggest that the immunodeficiency reported in LSIL patients could be due to the inability of specific cytotoxic T lymphocytes that for some unknown reason are present but unable to mount a response when challenged with their antigens, probably related to an *in situ* IL-2 production deficiency.

## Background

Evasion of the immune response by malignant cells is associated with the development of neoplastic diseases. Several mechanisms have been described that negatively regulate the immune system against foreign antigens. Among these mechanisms a bias in the secretion of cytokines produced by T cells towards the Th2 profile (IL-10, IL-4) as well as the inhibition of cytotoxic T cells (CTLs) has been proposed [1]. It has also been suggested that an increased activity of regulatory T cells may prevent an effective immune response against transformed cells and associated pathogens [2,3].

Cervical cancer (CeCa) is strongly associated to Human Papillomavirus (HPV) infection, and the progression of the disease depends on continuous viral replication and the eventual integration of the viral genome into the host genome [4]. The presence of specific T lymphocytes to HPV antigens in healthy subjects and in patients with different degrees of illness has been described [5]. The biological significance of these responses in healthy individuals has been interpreted as the mark left by successful virus elimination [6]; however, the presence of this response in patients with CeCa might indicate a poor ability of the lymphocytes to eliminate the virus. Specific T cell responses to various HPV proteins have been found, indicating that viral antigen recognition is a constant during the different phases of viral infection [5]. The presence of CD8 + IFNγ + lymphocytes is a normal occurrence since the early stages of viral infection [7]. However, in a number of cases their activity is unable to eradicate infected cells, which may contribute to the development of malignancy.

It is known that activation of CD8+ effector T cells requires the collaboration of CD4+ T cells, in particular for the generation of memory clones. In turn, the initial activation of naive CD4+ and CD8+ T cells depends on specific antigen presentation by activated dendritic cells. Antigen presentation and cellular effector activity during an antiviral response are regulated by cytokines such as IL-2, IL-12 and IFNγ. However, the instructive mechanisms that define the outcome of the antigen-presenting cells to T lymphocytes have not been fully elucidated [8]. In the case of HPV, the nature of antigen presentation and the resulting effector response is hampered by the fact that HPV does not have an active lytic phase, thus limiting the inflammatory process, which seems to be an important requirement for activation of antigen-presenting cells [9].

The purpose of this study was to evaluate the capability of T lymphocytes from Low-grade Squamous Intraepithelial Lesions (LSIL) patients to be activated by vaccine antigens. The participation of dendritic cells, CD8 + IFNγ cells and Treg cells during T cell activation was also determined. In addition, the production of Th1- and Th2-type interleukins by T cells stimulated by a CD3/CD28 agonist was quantified in an attempt to identify host factors associated with the deficient anti-HPV response known to be present in LSIL patients.

## Materials and methods

### Subjects and biological samples

Patients attending the Clinic of Dysplasias of the Cruz-Talonia Foundation were studied. Informed consent was obtained from all women included in this work. Human material was handled according to institutional experimentation and safety guidelines. The study population was selected from women referred for colposcopy due to abnormal cytology (n = 20), and women without a history of cervical abnormalities who attended the clinic for routine gynecological examination (n = 20). All women underwent cytological and histopathological analysis of colposcopy-directed samples. Cytology diagnosis was classified according to the Bethesda system [10]. Colposcopy-directed biopsies were obtained from acetowhite or normal tissue around the transformation zone. Tissue samples were placed in tubes containing sterile, contaminant-free phosphate-buffered saline and processed the same day for DNA isolation. Blood samples were obtained from either 20 LSIL patients, or 20 normal donors with normal cytology as control and a group of 5 young healthy women who were previously vaccinated with three doses of Gardasil® (Merck S&D, USA).

### HPV DNA detection by PCR

All reagents used for the isolation and amplification of DNA were purchased from Invitrogen, USA. Cervical biopsies were treated with proteinase K. DNA was extracted with phenol/chloroform and precipitated with ethanol. HPV DNA was amplified using the general primers MY09 (5’-CGTCCMARRGGAWACTGATC-3′) and MY11 (5′-GCMCAGGGWCATAAYAATGG-3′), which amplify a conserved 450-bp fragment from the L1 gene [11]. Genomic DNA (100 ng) was denatured by heating to 95 °C for 30 s. Annealing of primers was performed at 45 °C for 30 s and extension at 72 °C for 60 s. The cycle was repeated 30 times. Specific amplification of HPV-6, -11, -16, -18, -31, -33, -39, -45, -52, and −58 was achieved by using a set of primers for E7 specific PCR previously described in [12]. Genomic DNA (100 ng) was denatured by heating to 95 °C for 60 s. Annealing of primers was performed at 45 °C for 2 min and extension at 72 °C for 90 s. The cycle was repeated 40 times. To ensure DNA integrity, the β-actin gene was amplified for all samples.

### Lymphocytes proliferation assay

Serum from blood samples was separated from the cellular fraction by centrifugation. Serum samples were heat-inactivated by incubation at 57 °C for 30 min and then crio-preserved at −70 °C until use. The cellular fraction was mixed 1:1 v/v with PBS, loaded onto a 1.077 Histopaque gradient (Sigma-Aldrich, USA) and centrifuged to isolate the layer containing the peripheral blood leukocytes (PBL). 5×10^5^ PBL were seeded in 96-microwell plates (Corning, USA) in 200 μL of IMDM (Invitrogen, USA) supplemented with 10% of autologous serum (IMDM10S), either with medium alone, in the presence of 1 μL of Infanrix Hexa® (GSK Biomedicals, Belgium) corresponding to 6×10^-2^ IU of diphtheria, 8×10^-2^ IU of tetanus, 50 ng of pertussis, 20 ng of hepatitis B, and 0.16 IU of polio antigens, or in the presence of 1 μL of Cervarix® (GlaxoSmithKleen, Belgium) corresponding to 40 ng of each HPV-16/-18 L1 proteins, for 5 days. Thus, the assay consisted of three sets of samples: a) LSIL, b) normal, and c) young women vaccinated with Gardasil®. Each set of samples was challenged with Cervarix® or Infanrix®. We used as a control a culture in the absence of antigen. Then, 0.5 μCi of tritiated thymidine (Dupont NEN, USA) was added per well and incubated for another 16 hours. Finally the PBL cells were harvested and the incorporation of tritiated thymidine determined. The proliferation index corresponded to the ratio of: the cpm of cells incubated with antigen/cpm of control cells for each sample.

### Enzyme-linked immuno sorbent assay (ELISA)

Serum antibodies from patients with LSIL and from healthy donors were tested in ELISA. For this pourpose, immuno-plates (Costar-Corning,USA) were coated with 50 μL/well of Infanrix Hexa® or with 50 μL/well of Cervarix® diluted 1:20 in PBS and incubated at 4 °C overnight. Plates were washed and non-specific binding sites were blocked with 2% BSA (Research Organics, USA) for 2 hr at 37 °C. Then 50 μL of blood serum diluted 1:500 were added to the plate and incubated for 2 hr at 37 °C. 50 μL of alkaline phosphatase-conjugated goat-anti human IgG (Invitrogen, USA) diluted 1:5000 were added and incubated for 1 hr at 37 °C. Alkaline phosphatase substrate (Sigma-Aldrich, USA) diluted in a 10% (w/v) diethanolamine solution (Sigma-Aldrich, USA) was finally added to the plates. The absorbance was read at 405 nm in an ELISA plate reader (Molecular Devices, USA). The final values were obtained after subtracting the non-specific absorbance reactivity obtained in wells with saline to the reactivity obtained in wells with antigen.

### Determination of CD4 + CD25 + FoxP3+ cells by flow cytometry

5×10^5^ PBL were incubated with 1 μL of antiCD4/PerCP (R&D Systems, USA) and 1 μL of antiCD25/PE (R&D Systems, USA) at 4 °C for 30 min, washed twice and subsequently permeabilized and labeled with antiFoxP3/APC (eBioscience, USA) and isotypes involved. After three final washes, the cells were fixed with 2% paraformaldehide (Sigma-Aldrich, USA) and the percentage of CD4 + CD25 + FoxP3+ cells was determined by flow cytometry (FacsAriaII, BD, USA).

### Isolation and maturation of monocytic-derived dendritic cells

PBL were fractionated by adherence to plastic dishes during 2 hr at 37 °C. The adherent fraction was then cultured for 4 days in IMDM-10S supplemented with 30 ng/ml of rhGM-CSF (R&D Systems, USA) and 20 ng/mL of rhIL-4 (R&D Systems, USA). The cells were then harvested, washed and incubated for one hour with 5 μL/mL of Cervarix® at 37 °C. Finally the cells were kept in culture for two days more in IMDM-10S with rhGM-CSF, rhIL-4 and 20 μg/mL of poly I:C (Sigma- Aldrich, USA), to induce their maturation, either in the presence or in the absence of 1μL of Cervarix®.

### Lymphocytes proliferation induced by dendritic cells assay

PBL from two patients (one positive for HPV-16 and other for HPV-18) were induced to proliferate in the presence of L1 from HPV-16 and −18 processed and presented by either a): monocytic cells or b): dendritic cells. For this purpose: a) PBL were incubated with 2 μM of carboxyfluorescein (CFSE) (Sigma-Aldrich, USA) during 15 min at room temperature (RT). After three washes with IMDM10S, 5x10^5^ cells were incubated in 200 μL of IMDM10S during 5 days either with 1 μL of Cervarix®, or in absence of antigen, as a control. b): PBL were labeled with CSFE during 15 min, RT, washed three times and then either incubated with autologous monocytic-derived dendritic cells loaded during maturation with 1μL of Cervarix®, or with autologous dendritic cells used as a control. After 5 days the cells were harvested and stained with 1μL of CD4/PE antibodies (R&D Systems, USA) and 1 μL of CD8/APC antibodies (R&D Systems, USA) at 4 °C during 30 min. The decrease in the carboxyfluorescein label was assessed by flow cytometry, and used as an indicator of proliferation.

### Determination of CD8 + IFNγ + cells induced with PHA

5×10^5^ PBL were seeded in 96-microwell plates (Corning, USA) in 200 μL of IMDM10S, in the presence of phytohemagglutinin (PHA) (Invitrogen, USA) dilute 1:100 as the final concentration, or in absence of PHA as a control, for 5 days. After this time, the cells were harvested and stained with 1 μL of CD8/APC and 1 μL of IFNγ/FITC (R&D Systems, USA), for 30 min at 4 °C, and finally evaluated in a FACSAria II cytometer (BD, USA). The percentage of CD8 + IFNγ + cells was determined for each sample based on the total cell number recovered in the culture.

### Quantification of cytokines

5×10^5^ PBL were incubated in 200 μL of IMDM-10S with 2.5 μL of antiCD3/CD28 beads (Dynabeads CD3/CD28 Human T-Activator, Invitrogen, USA). After 4 days, the conditioned medium was collected and stored at −70 °C. The concentration of cytokines was determined by flow cytometry according to manufacturer’s instructions (Cytometric Bead Array: Human Th1/Th2 cytokine kit, BD Biosciences, USA). Plasma cytokines levels were determined with the same Th1/Th2 cytokine kit from peripheral blood samples, after separation of the cellular fraction, also according to manufacturer’s instructions.

### Statistical analysis

All samples were tested in triplicate and the numerical data are expressed as an average of the values obtained. The non-parametrical Man-Whitney *U* test was used to compare the mean of the pertinent groups. A *P <0.05* was considered statistically significant.

## Results

### Lymphocytes from LSIL patients had a significantly lower response to vaccine antigens than normal donors

**Figure 1:**
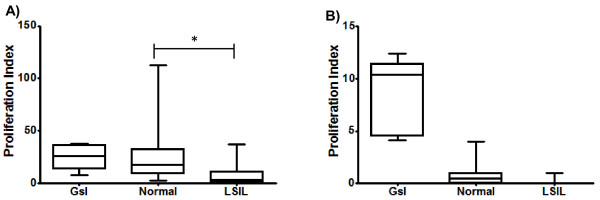
**Response of PBL from LSIL patients and normal donors to already known antigens**. **A**) PBL from LSIL patients (LSIL) (n = 20), normal donors (Normal) (n = 20), and women vaccinated with Gardasil® (Gsl) (n = 5), were incubated with antigens from Infanrix Hexa®, a vaccine containing antigens of diphtheria, tetanus, pertussis, hepatitis B and poliovirus, which is administered during childhood. **B**) PBL from LSIL patients (LSIL) (n = 20), normal donors (Normal) (n = 20), and women vaccinated with Gardasil® (Gsl) (n = 5) were incubated with Gardasil® a recombinant vaccine containing capsid antigens from HPV16, and HPV18. Proliferation was determined by means of thymidine incorporation after 5 days incubation, and the proliferation index corresponded to cpm in the presence of antigen/cpm control without antigen. A statistical significant difference was found between LSIL and normal groups (* *p* < 0.005).

After clinical and molecular diagnosis, subjects were divided into two groups: the LSIL group (n = 20; mean age: 26.65 years, range: 18–35 years), and the normal donor group (n = 20; mean age: 37.93 years, range: 22–46 years). HPV analysis showed that five LSIL samples were positive for the presence of HPV DNA. Genotyping of these positive samples demonstrated the presence of individual infection by HPV types 6, 11, 16, 18 and 58. These samples were included in the set of 20 that formed the group named LSIL in the assays. In order to assess the presence of cells reactive to well known antigens, PBL from LSIL patients, women vaccinated with Gardasil®, and normal controls were incubated with Infanrix Hexa®, a vaccine that is administrated in Mexico as a part of the national vaccination campaign. As expected, cells from normal controls and women vaccinated with Gardasil® were able to proliferate in the presence of the antigens contained in Infanrix®. In contrast, PBL from LSIL patients showed a response to the vaccine that was significantly lower than the detected in normal donors (Figure 1). To demonstrate that the response was directed against an already known antigen, PBL were also incubated with Cervarix®, a vaccine that contains antigens from HPV16 and HPV18. In Mexico, administration of Cervarix® is basically restricted to private practice; therefore women included in this work have not been vaccinated with this vaccine. As shown in Figure 1, cells from women vaccinated with Gardasil® proliferated as a response to the viral antigens contained in Cervarix®. Interestingly, cells from both HPV-infected and non-infected LSIL women showed a negative response, that was similar to that detected in normal controls. These results suggest the existence of a deficiency on the response of T cells from patients with LSIL.

### Serum antibodies to vaccine antigens are found in LSIL patients and normal donors

**Figure 2:**
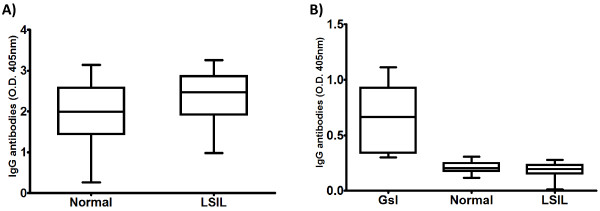
**A) Seroreactive response of normal donors (Normal) and LSIL patients (LSIL) to Infanrix Hexa® antigens.** Analysis of the presence of IgG antibodies specific to Infanrix Hexa® antigens (diphtheria, tetanus, pertussis, hepatitis B and poliovirus) was determined by ELISA. Infanrix Hexa antigens were diluted 1:20 to cover plate and serum samples were diluted 1:500 to assay their seroreactivity. No statistically significant difference between LSIL and the normal group was found. (**B**) Seroreactive response of normal donors (Normal), LSIL patients (LSIL) and women vaccinated with Gardasil® to Cervarix® antigens. Analysis of the presence of IgG antibodies specific to Cervarix® (L1 protein from HPV-16 and −18 capsids) was determined by ELISA. Cervarix® antigens were diluted 1:20 to cover plate and serum samples were diluted 1:500 to assay their seroreactivity. No statistically significant difference between LSIL and the normal group was found. O.D.: Optical density.

Our results showed a decreased T cell response in LSIL patients. Thus, to determine if the antibody response was also diminished, we evaluated the presence of antibodies to Infanrix® in sera from LSIL patients and normal controls by ELISA using Infanrix® as target antigens. We detected similar antibody levels in serum samples from LSIL patients and normal donors without HPV infection (2.35 ± 0.66 and 1.96 ± 0.76, respectively) (Figure 2). These results suggest that the difference in T cell responses between LSIL patients and normal controls is not associated with a reduced function of B lymphocytes. Additionally, we tested the presence of antibodies specific to L1 from HPV-16 and −18 in the sera of LSIL patients and normal donors, and obtained a minimal response; in contrast, we found those specific antibodies in sera from young women vaccinated with Gardasil® (composed by L1 from HPV-6, -11, -16 and −18).

### Similar levels of CD4 + CD25 + FoxP3+ lymphocytes were present in PBL from LSIL patients and normal donors

**Figure 3:**
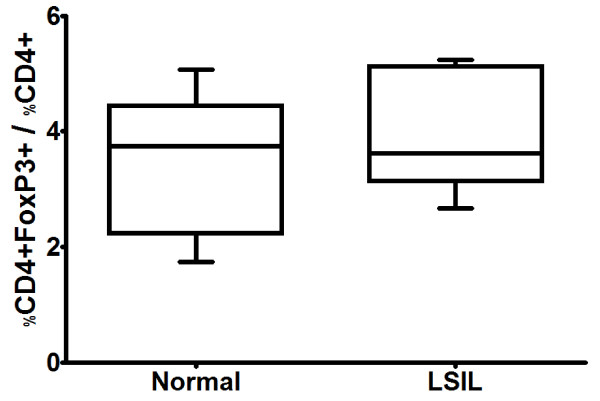
**Characterization of the presence of cells with regulatory phenotype in peripheral blood lymphocytes from patients with LSIL (LSIL) and donors without HPV (Normal).** Peripheral blood mononuclear cells isolated from healthy donors and from LSIL patients were analyzed without previous stimulation for surface expression of CD4 and CD25 and intracellular expression of FoxP3. CD4 + CD25 + FoxP3+/CD4+ cells ratios were determined for each sample. No statistically significant difference between LSIL and the normal group was found.

In order to evaluate if the diminished T cell response in LSIL patients is associated with the presence of Treg cells, we evaluated the levels of CD4 + CD25 + FoxP3+ cells. No significant difference was found in the proportion of CD4 + CD25 + FoxP3+ cells in PBL from LSIL patients (3.96 ± 1.00) and normal controls (3.51 ± 1.18) (Figure 3). These results suggest that Treg cells do not account for the deficient antigen recognition by PBL cells in LSIL patients.

### PHA induces the production of IFNγ by CD8+ lymphocytes from LSIL patients

**Figure 4:**
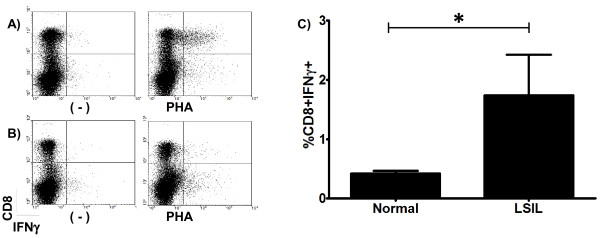
**Proliferation of CD8 + IFNγ + cells from LSIL patients (LSIL), and normal donors (Normal) after stimulation with phytohemagglutinin (PHA).** PBL from LSIL patients were cultured for 3 days in the presence of PHA, and then analysed for surface CD8 and intracellular IFNγ expression. The proportion of CD8 + IFNγ + stimulated by PHA were evaluated by flow cytometry. **A**) shows a representative plot obtained from PBL of a LSIL patient incubated with phytohemagglutinin (PHA) and in absence of PHA, as a control (−). **B**) shows a representative plot obtained from PBL of a normal donor incubated with phytohemagglutinin (PHA) and in absence of PHA (−). Bars in **C**) depict the mean of CD8 + IFNγ + percentages for each set of samples. Error bars indicate the standard error of the mean. A statistically significative difference was found between the groups with a *p* < 0.05*.

Our results indicate that the lack of lymphocyte response in LSIL patients is not due to negative regulation by Treg cells. Therefore, we next investigated whether CD8 + IFNγ + lymphocytes, which are known to play an active role during anti-viral responses, can be activated in these patients. PBL from LSIL patients and normal controls were stimulated with PHA. The proportion of CD8 + IFNγ + cells was determined by flow cytometry. We observed a high proportion of CD8 + IFNγ + cells in the PBL from LSIL patients, while almost no lymphocytes of this phenotype were detected in the normal donors (Figure 4). These results suggest the presence of inducible CD8 + IFNγ + cytotoxic lymphocytes in LSIL patients.

### Lymphocytes from LSIL patients were strongly induced to proliferate in the presence of dendritic cells

**Figure 5:**
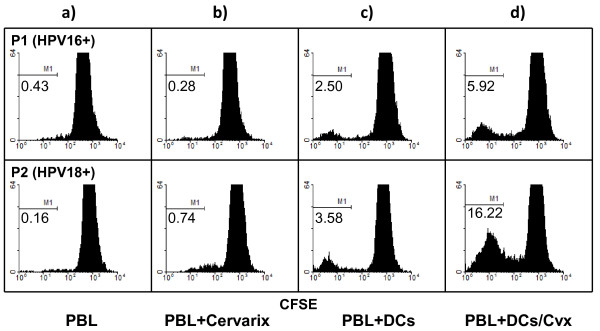
**Proliferation of PBL from LSIL patients in response to HPV antigens presented by monocytic (a,b) and by dendritic cells (c,d).** PBL from two LSIL patients, infected by HPV-16 (P1), and HPV-18 (P2) were incubated with Cervarix®, that contains capsid antigens of HPV-16, and HPV-18, to evaluate the induced proliferation. Carboxifluorescein (CFSE) labeled PBL from patients P1 and P2 were incubated in the presence of soluble Cervarix® (PBL + Cervarix) (**b**), and PBL alone were used as a control (PBL) (**a**). CFSE labeled PBL from patients P1 and P2 were cultured with autologous dendritic cells (PBL + DCs), previously “loaded” with Cervarix® (**d**), or with autologous unloaded dendritic cells as a control (PBL + DCs/Cvx) (**c**). The percentage of decrease in carboxiflourescein label, as an indicator of cell proliferation is shown under the bar (M1) of each histogram.

Our results indicate that subpopulations of inducible CD8 + IFNγ + cytotoxic lymphocytes are present in LSIL patients. However, it is known that activation of CD8+ cells depends on specific antigen presentation by activated dendritic cells. Therefore, we next evaluated whether lymphocytes from LSIL patients could be activated to proliferate as a response to already known viral antigens presented by autologous dendritic cells. PBL from two LSIL patients, were co-cultured with dendritic cells or dendritic cells previously “loaded” with Cervarix®. The first patient was infected by HPV18; the second one was infected by HPV16, as demonstrated by PCR. Proliferation of PBL was determined. As shown in Figure 5, PBL from the patients were able to proliferate in the presence of HPV antigens alone. Co-culture with dendritic cells induced a modest increment in PBL proliferation. However, activation by pre-loaded dendritic cells induced a dramatic increase in PBL proliferation in both patients. These results indicate that PBL from LSIL patients are able to recognize and proliferate as a response to already known viral antigens, but the response completely depends on the presentation of antigens by activated dendritic cells.

### Lymphocytes from LSIL patients produce lower levels of IL-2 than those from normal donors

**Figure 6:**
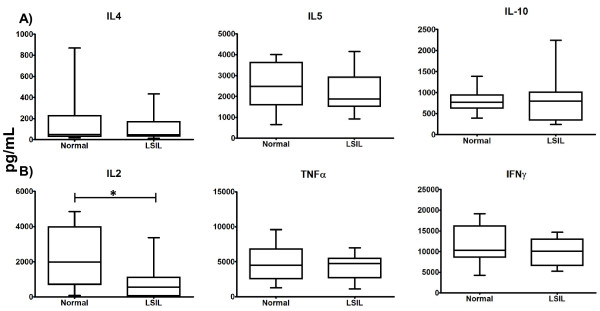
**Cytokines production after stimulation of antigenic receptor of PBL from patients with LSIL (LSIL) and normal donors without HPV (Normal).** PBL from LSIL patients and normal donors were stimulated in culture with CD3/CD28 activating beads. After 5 days in culture, the concentration of: A) Th2 (IL-4, IL-5, IL-10) and B) Th1 (IL-2, TNFα, IFNγ) cytokines in supernatants were assayed by mean of a Cytometric Bead Array. A statistically significant difference was found for IL-2 production (* *p* < 0.05).

Our results have shown that PBL from LSIL patients can be specifically activated by dendritic cells presenting an already known viral antigen. Inasmuch as antigen presentation and cellular effector activity during a viral infection are regulated by cytokines, we evaluated the levels of serum IL-2, IL- 4, IL-6, IL-10, TNFα and IFNγ from LSIL patients and normal controls. No differences in concentration of any of the circulating cytokines tested were found between LSIL patients and normal controls (data not shown). Thus, we decided to explore the production of these cytokines by LSIL and normal control PBL after the induction of proliferation by an agonist of CD3/CD28. We observed that the PBL from LSIL patients and normal controls produced and secreted similar levels of IL-4, IL-5, IL-10, IFNγ, and TNFα. However, production of IL-2 was significantly reduced in LSIL patients (Figure 6). These results seem to indicate that production and secretion of the main regulator of T cell activation, IL-2, is highly compromised in LSIL patients.

## Discussion

Progression of precursor lesions (LSIL and HSIL) towards cervical cancer has been associated with persistent infection by oncogenic HPV types, along with local and systemic immune abnormalities, which may lead to impaired function of the T cell response. Most LSIL are expected to undergo a spontaneous regression [13], which is normally associated with the development of an efficient cellular response to HPV antigens [14]. In the present work we have shown that LSIL patients exhibit a deficient T cell response, not only to HPV antigens, but also against various types of antigens, and that the lack of response is unrelated with a current HPV infection. Lymphocytes from LSIL patients showed significantly less proliferation as a response to already known vaccine antigens and to HPV-derived antigens, than those from normal donors, even when the concentration of specific antibodies to these antigens were similar for both groups; nevertheless we consider worthwhile to increase the number of samples in the near future to generalize our results.

Specific T cell response to cancer cells and viruses is tightly regulated by regulatory CD4+ cells (Tregs) expressing fork-head box (Fox)-P3 (CD4 + CD25 + FoxP3+). Increased frequencies of circulating Tregs have been reported in various solid tumors [15], including cervical cancer [2,3]. Here we found that CD4 + CD25 + FoxP3+ regulatory cell numbers are not increased in LSIL patients. This observation is in agreement with a previous report showing a similar frequency of circulating CD4+ Tregs in women with CIN0 and patients with CIN1/2 [3] and with other report, where the higher levels of mucosal FoxP3 expression were shown in the CIN2/3 group [16]. Accordingly, we did not find a correlation between the lack of proliferation and IL-2 secretion with an increased presence of CD4 + CD25 + FoxP3+ cells in our samples.

IFNγ-producing T cells play a crucial role in the elimination of tumor cells [17]. It has been demonstrated that although HPV-specific activated CD4+ and CD8+ cells are detected in tumor-infiltrating lymphocytes (TIL) and tumor-draining lymph node cells (TDLNC) of most cervical cancer patients, only a few IFNγ-producing cells are found in these patients, suggesting that the T cells are functionally inactive [18]. In our work, we found that stimulation of PBL from LSIL patients with PHA resulted in a significantly greater proportion of CD8 + IFNγ + cells compared with those obtained in healthy donors, which suggest the existence of subpopulations of responder cells with a limited state of activation in the earliest stages of pre-malignant cervical lesions. In order to determine whether these clones correspond to HPV-specific T cells a more thorough characterization of these populations to identify their antigenic specificity and their activation status is needed. Coincidently, Lee and colleagues [19] found that CD8+ lymphocytes from LSIL or ASCUS patients stimulated with *Staphylococcus* enterotoxin B had a deficiency in the production of IL-2, but not of IFNγ, suggesting that the effector ability of CD8+ cell is not affected, although this population cannot be expanded.

We found that lymphocytes from HPV16- and HPV18-associated LSIL were not able to proliferate in the presence of soluble viral antigens derived from HPV-16 and −18 L1 proteins, even when they did it against Infanrix® derived antigens. However, a strong T cell response was observed when the antigens were presented by dendritic cells. A similar observation was made in cervical cancer, where stimulation of TIL and TDLNC with their cognate antigen in the presence of commonly used Toll-like receptor ligands significantly enhanced the effector T-cell function. Therefore, it is possible the presence of specific T cells but with limited activation status due to lack of appropriate stimulation since the early stages of infection with HPV to malignant stages. The mechanisms leading to this state of partial activation by T lymphocytes remain to be resolved, but may be associated with the limited antigen presentation and absence of inflammation that occurs in the early stages of infection with HPV [9]. In this sense, several lines of evidence show that failure in the presentation of antigens by dendritic cells, may contribute to the development of tumors [20-22].

It has been considered that an effective immune response to HPV is based on a Th1-type reaction, which involves cytokines such as IL-2, IL-12, TNFα and IFNγ. It has been observed that the elimination of the virus is preceded by the expression of Th1 cytokines in the absence of Th2-type cytokines [23], and given the antagonistic nature of Th1 and Th2 functions, it is assumed that infected cells are protected from immune response when a Th2 pattern is present, with a significant role for IL-10 [24]. However, in more advanced stages, including cervical cancer, it is possible to detect a large production of cytokines of both profiles, questioning the role of Th2 profile in cervical cancer development [25]. We found that IL-2 secretion induced by stimulation of T cell antigen receptor was significantly lower in PBLs from LSIL patients in comparison with those of healthy donors. These results are consistent with the notion that Th1 profile is necessary for the resolution of infection, as IL-2 production shows a deficiency that is reflected beyond the local level, to the periphery. However, we did not find a difference in the production of IFNγ, IL-10, and TNFα between PBL from LSIL and normal donors. It has been previously reported a decreased production of IL-2 by PBL from HPV infected subjects, stimulated *in vitro* with both HPV specific and unrelated antigens, as well with polyclonal stimuli [19,26,27]. In addition, it has been demonstrated that CD8+ T cells from LSIL patients have a deficiency in the production of IL-2 when they are stimulated with *Staphylococcus* enterotoxin B [19]. In all these cases, a common feature was a direct relationship between the degree of the IL-2 deficiency and the lesion progress. One proposed explanation for this deficiency has been a depletion of the immune system as a result of inflammation due to HPV and other pathogens that associate with LSIL and HSIL [19]. Other possible causes of inhibition of proliferation and IL-2 production without impairing effector function in T lymphocytes could be associated with metabolic process, for example thiol-sensitive pathways, involving IL-2 [28]. However, the precise events that are lead to this deficiency have not been fully determined.

The above results suggest a key role for IL-2 in the resolution of the infection and elimination of tumors. IL-2 concentration decreases with advancing stages of the disease, when the production of other cytokines show a new increase in the most advanced stages, probably as a result of enhanced inflammation [29]. In this respect we have previously shown that the presence of IL- 2 receptors on cancer cells could compete for the IL-2 present *in situ* depleting the concentration of this growth factor that is absolutely needed to activate cytotoxic cells [30]. It is known that an adequate memory CD8+ T cells setting is highly dependent on helper CD4+ T cell function, and IL-2 has been proposed as a signal required during priming for a secondary expansion of CD8+ T cells [31], particularly in the case of chronic infections [32]. Therefore, the restricted activation of CD8+ T cells detected in the blood of LSIL patients could be explained by the deficiency of IL-2 observed in these leukocytes. In addition, CD4+ helper activity needs dendritic cells for efficient priming of T CD8+ cells [33] which in turn are known to be producers of IL-2 [34].

In this work we present evidence that LSIL patients show a deficiency in the T cell response, which is not associated with an increase in T regulatory cells or an increase in Th2 inhibitory cytokines. We also showed that PBL from LSIL patients can be activated *in vitro* to recognize HPV, and significant amounts of CD8 + IFNγ + cells can be obtained from LSIL patients after mitogenic stimulation. However, LSIL patients are unable to mount a specific response when challenged with those antigens unless they are presented by autologous dendritic cells. The fact that PBL from those patients were poor producers of IL-2 suggests that a deficiency *in situ* of IL-2 could be a mechanism by which infected cells escape immune surveillance and thus create the conditions for the subsequent expansion of malignant clones.

## Abbreviations

HPV: Human papillomavirus; PBL: Peripheral blood leukocytes; LSIL: Low-grade squamous intraepithelial lesions; HSIL: High-grade squamous intraepithelial lesions; CTL: Cytotoxic T lymphocytes; CeCa: Cervical cancer; Th: T helper lymphocyte; Tregs: T regulatory cells; DCs: Dendritic cells; CIN: Cervical intraepithelial neoplasia; TcR: T cell receptor; PHA: Phytohemagglutinin.

## Competing interests

The authors declare that they have no competing interests.

## Authors’ contributions

JHM conceived of the study and wrote the manuscript. LRZ performed the ELISA assays and writing of paper. AMG participated in proliferation assays and as well as in discussion of results. BWS participated in discussion of results and revision of the manuscript. FCT and OCC obtained tissue samples and participated in the determination of HPV infection. MCZO collected peripheral blood samples of the patients and normal donors. LCBA and PRA participated in dendritic cells generation and cytometry interpretation. AKCR and MVLH performed cytokines and Tregs determination. MLMG participated in the design and coordination of the study. All authors read and approved the final manuscript.
